# Supplementary material for: Harnessing C/N balance of Chromochloris zofingiensis to overcome the potential conflict in microalgal production
Source: Commun Biol. 2020 Apr 23;3:186. doi: 10.1038/s42003-020-0900-x (PMC7181789; doi:10.1038/s42003-020-0900-x)
Supplement: Supplementary file 1 — Supplementary Information [file 42003_2020_900_MOESM1_ESM.pdf]

1. Supplementary Methods

1.1 Determination of amino acid composition

Microalgal cells were collected and wished twice by centrifugation at 5,000 rpm for 5min. The cell pellet was then hydrolyzed by hydrochloric acid (6 mol L^{-1}) at 110°C for 12 h. The hydrolysates were analyzed by a HPLC- MS/MS system (Shimadzu LC20AD-API 3200MD TRAP). Three replicate measurements were carried out.

1.2 Determination of fatty acid composition

The cell pellet from 10 mL culture was extracted by 2 mL chloroform and 1 mL methyl alcohol, and oscillated for 30 min. Then 0.75 mL sodium chloride (0.75%) was added into the mixture, followed by centrifugation at 5,000 rpm for 5 min. After centrifugation, the chloroform layer methylated to fatty acid methyl esters (FAMEs) by incubating with 1% sulphuric acid in methanol. The FAMEs were analyzed by a gas chromatography-mass spectrometry (GC-MS) (Shimadzu, Kyoto, Japan) equipped with an Rtx-2330 capillary column (30 m × 0.25 mm × 0.25 μm) (Restek, Guangzhou, China). Three replicate measurements were carried out.

1.3 Determination of RNA and DNA

For determination of RNA, microalgal cells were collected by centrifugation at 4°C. The pellet was washed 3 times by 400 μL pre-cooled perchloric acid at 0.7 mol L^{-1}. Then 400 μL potassium hydroxide was added to digestion at 37°C for 60 min. After cooled at a room temperature, the solution was neutralized by 133 μL perchloric acid (3 mol L^{-1}). The supernatant was collected and the precipitate was dissolved by

533μL perchloric acid (0.5 mol L^{-1}). The supernatants were combined and diluted to 2 mL and then centrifuged at 10000 g to remove impurities. Finally, the RNA concentration was measured at 260 nm. Three replicate measurements were carried out.

For determination of DNA, microalgal cells were collected by centrifugation and wished twice by magnesium chloride (1mol L^{-1}). Then, 5 mL pre-cooled perchloric acid (0.25 mol L^{-1}) was added for 30 min on ice. The precipitate was dissolved by 4 mL perchloric acid (0.5 mol L^{-1}) at 70°C for 15 min. The supernatant was collected and the precipitate was incubated by 3 mL perchloric acid (0.5 mol L^{-1}) twice. The supernatants were combined into 10 mL and DNA was measured by diphenylamine method. Three replicate measurements were carried out.

2. Supplementary Figures

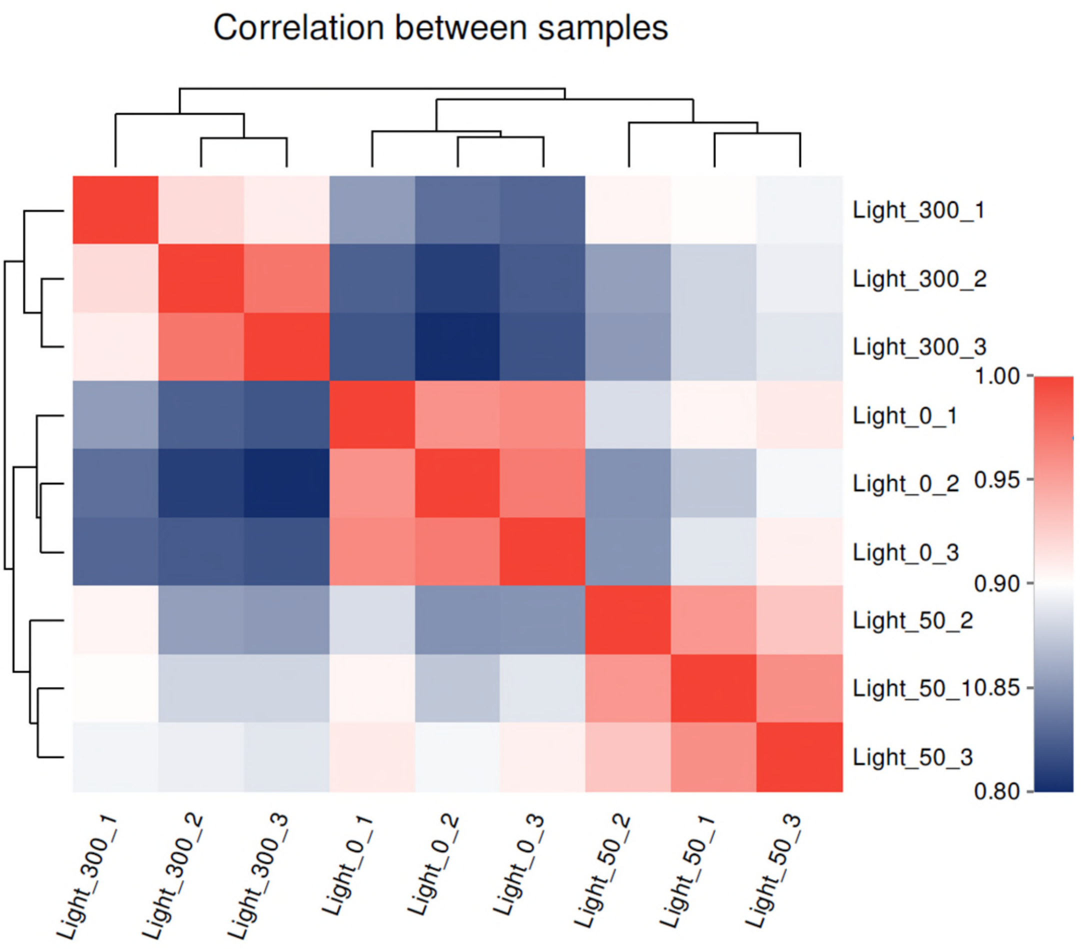

Supplementary Figure 1. Spearman correlation of transcriptomes among different biological samples.

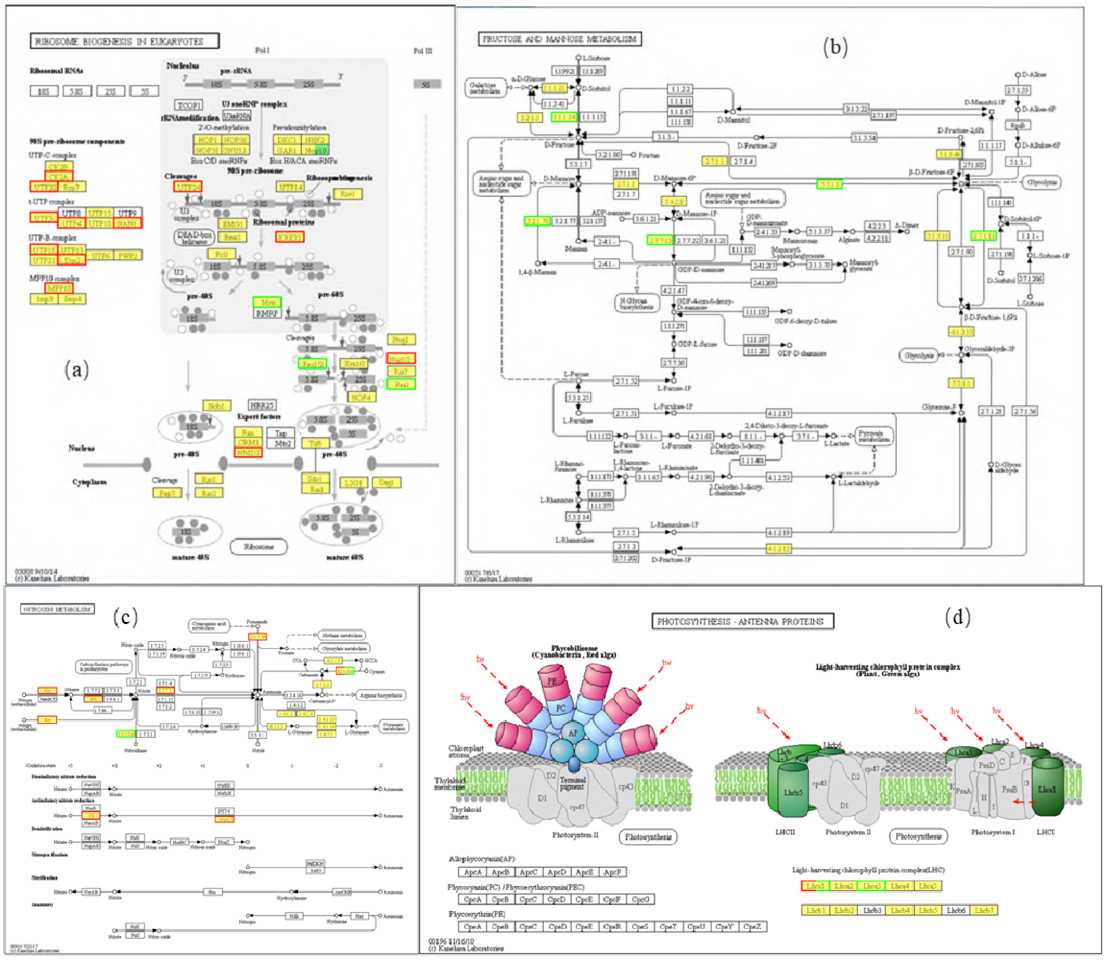

Supplementary Figure 2. Transcriptiome analysis of *C.zofingiensis* comparing under 50 μE m^{-2} s^{-1}, 0 μE m^{-2} s^{-1} in translation (a), carbohydrate metabolism (b), nitrogen metabolism (c) and photosynthesis-antenna proteins (d).

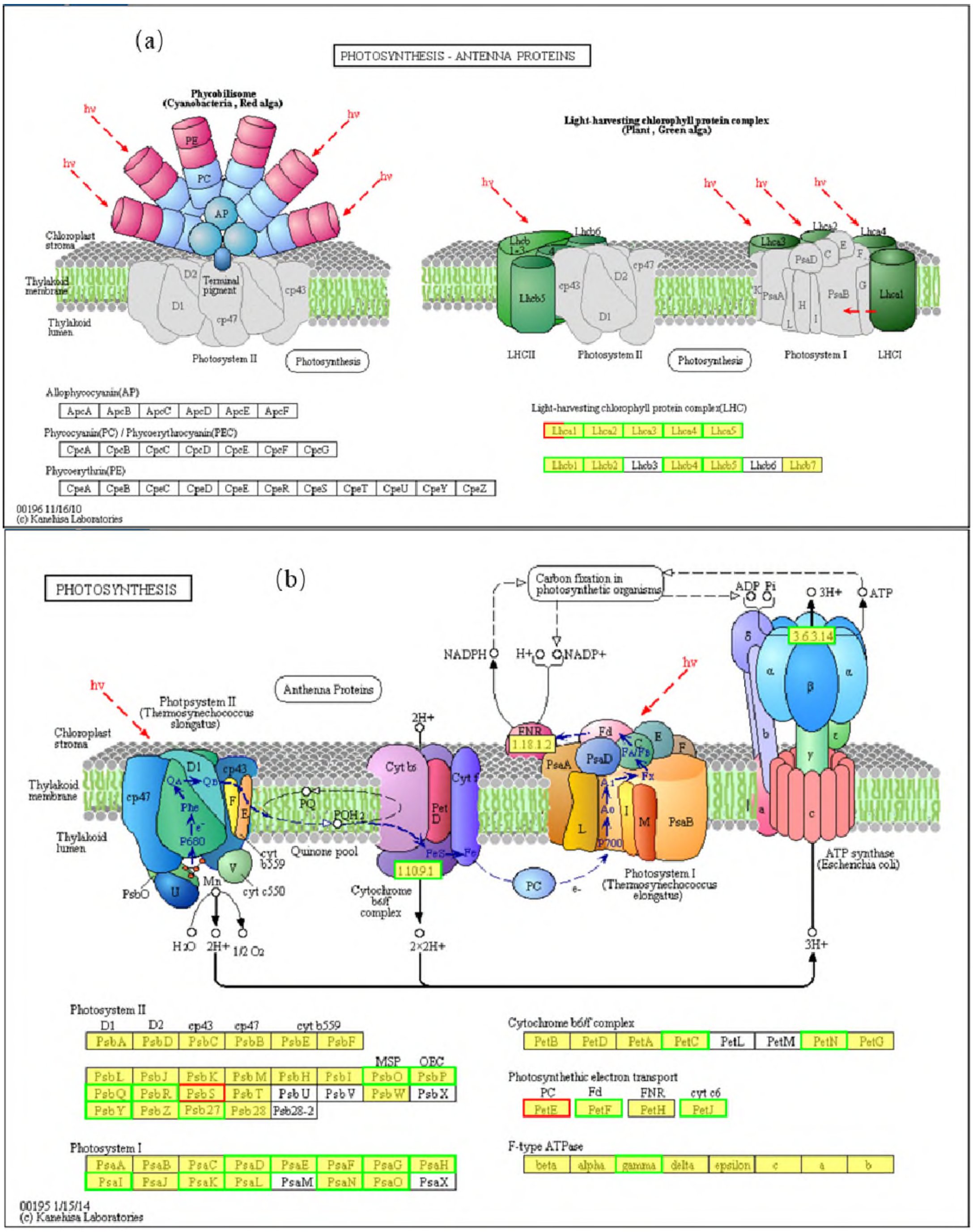

Supplementary Figure 3. Transcriptiome analysis of *C.zofingiensis* comparing under 50 μE m^{-2} s^{-1}, 300 μE m^{-2} s^{-1} in photosynthesis-antenna proteins (a) and photosynthesis (b).

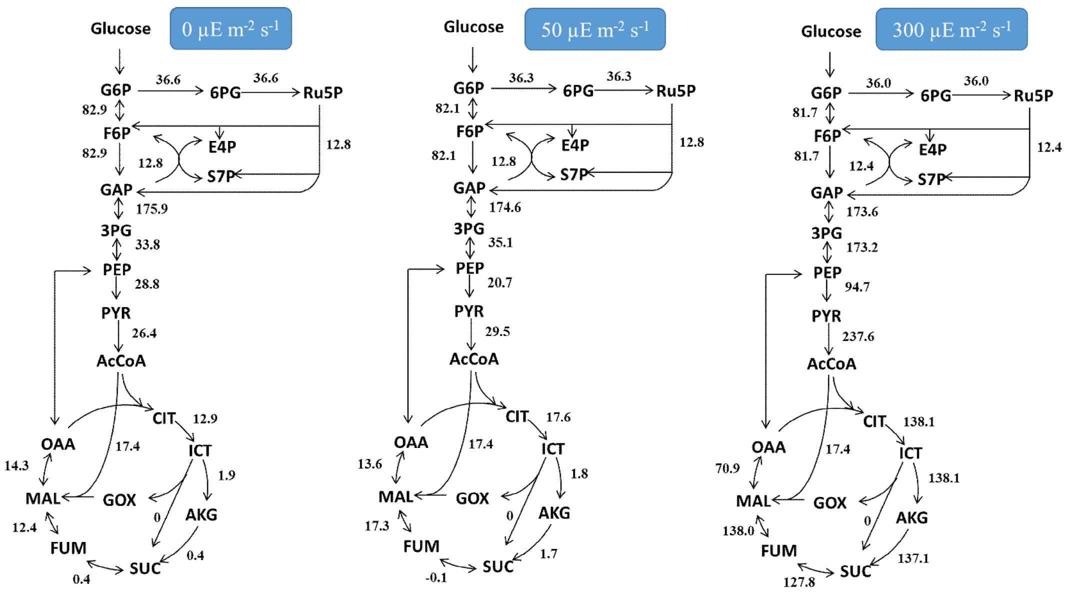

Supplementary Figure 4. Central carbon metabolism of *C.zofingiensis* comparing under 0, 50 and 300 µE m^{-2} s^{-1}.

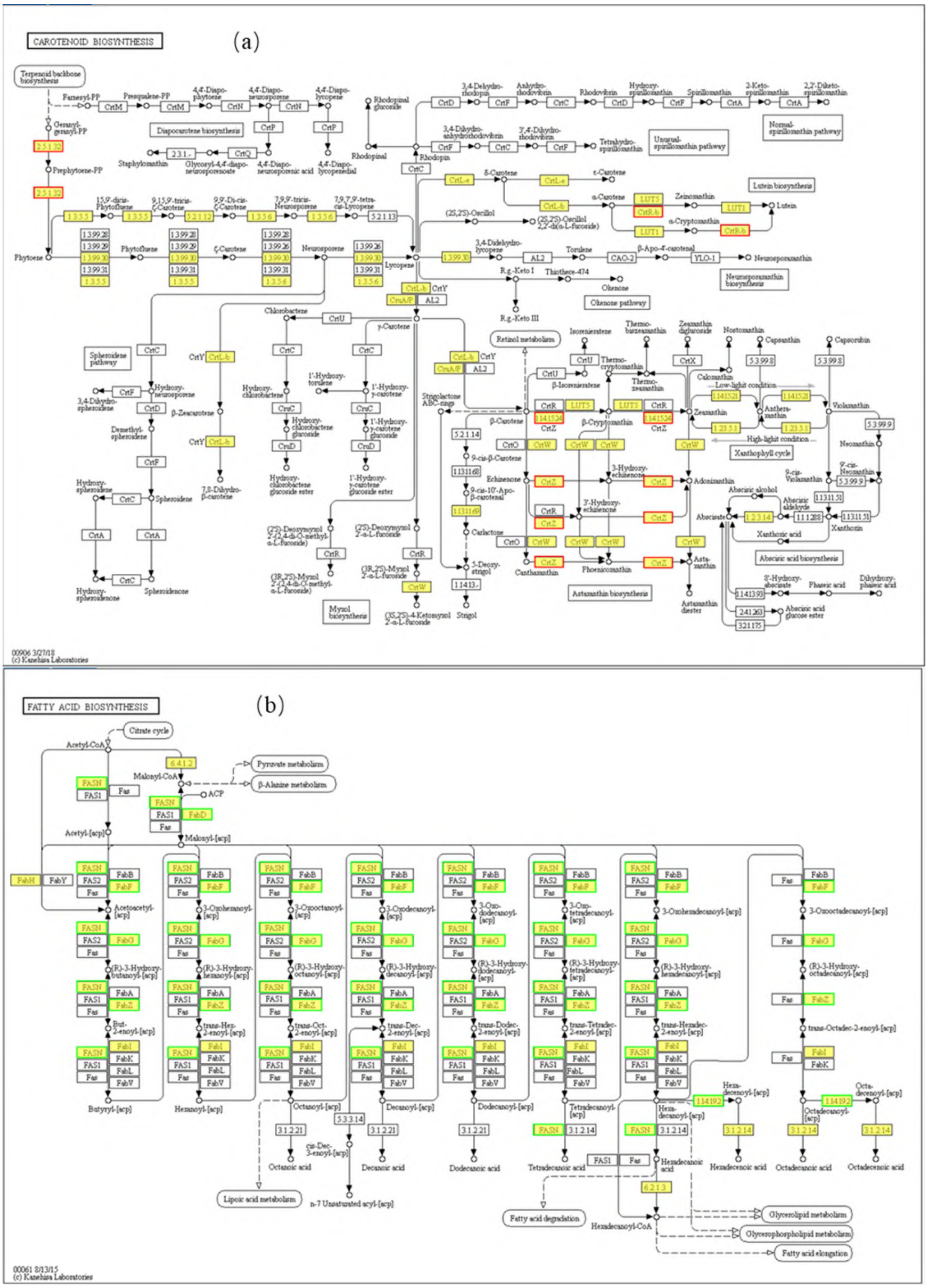

Supplementary Figure 5. Transcriptiome analysis of *C.zofingiensis* comparing under 50 μE m^{-2} s^{-1}, 0 μE m^{-2} s^{-1} in carotenoid biosynthesis (a) and fatty acid biosynthesis (b).

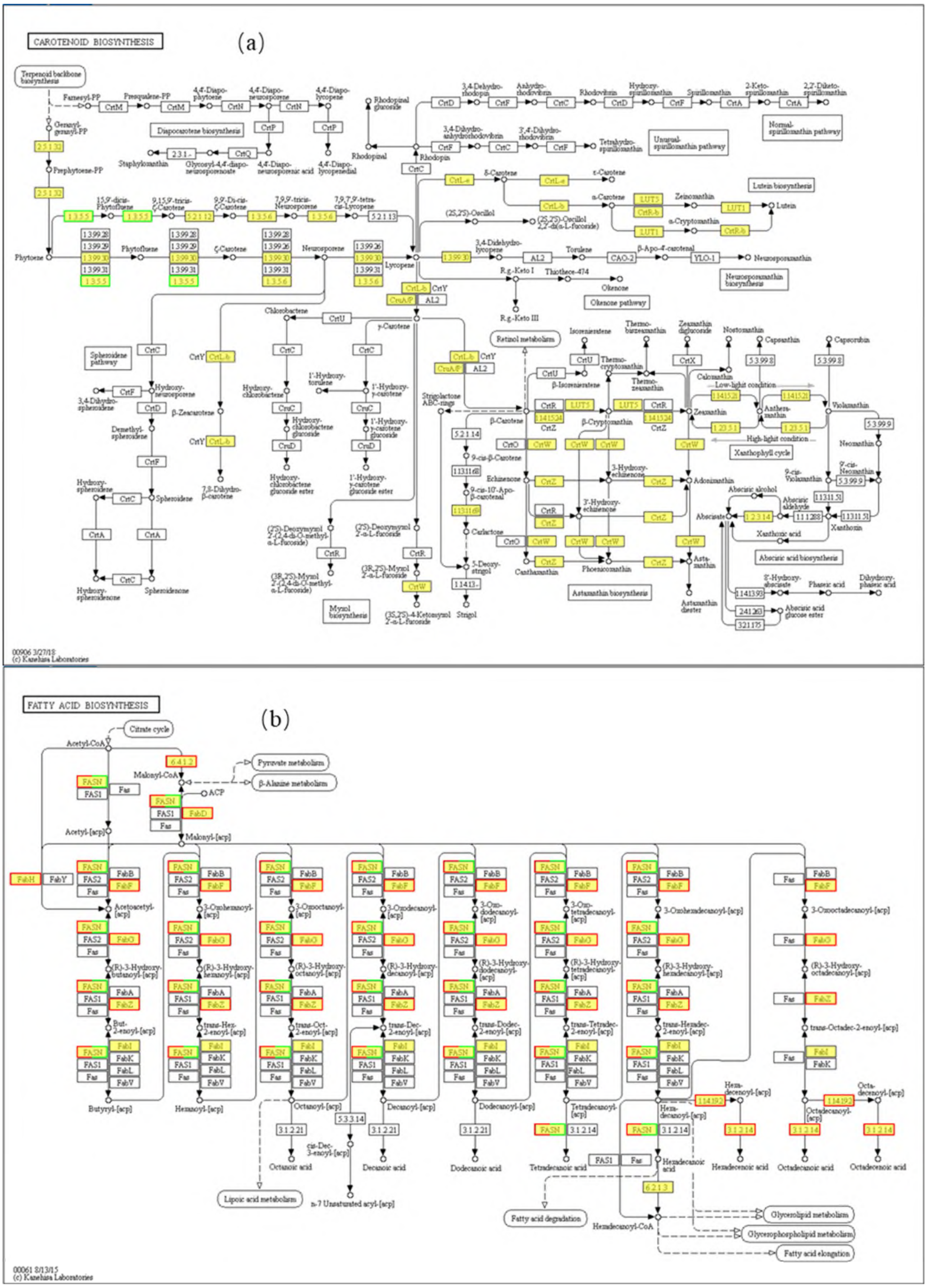

Supplementary Figure 6. Transcriptiome analysis of *C.zofingiensis* comparing under 50 μE m^{-2} s^{-1}, 300 μE m^{-2} s^{-1} in carotenoid biosynthesis (a) and fatty acid biosynthesis (b).

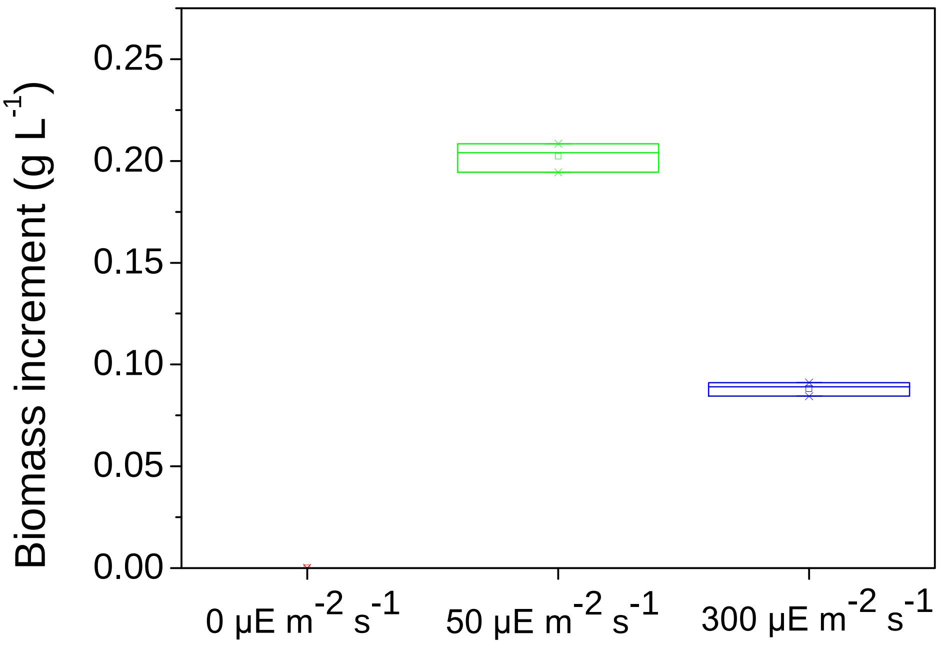

Supplementary Figure 7. The contribution of CO_2 to biomass concentration under different light intensities.

3. Supplementary Tables

Supplementary Table 1. Nucleotide composition of DNA and RNA in *C. zofingiensis*

	DNA	RNA
A	0.245	0.245
T(U)	0.245	0.245
G	0.255	0.255
C	0.255	0.255

Supplementary Table 2. Biomass formation of *C. zofingiensis* under different light intensities.

Coefficient	0 μE m^{-2} s^{-1}	50 μE m^{-2} s^{-1}	300 μE m^{-2} s^{-1}
rCTP	0.049637	0.049535	0.032903
rGTP	0.049637	0.049535	0.032903
rATP	0.04769	0.047592	0.031613
rUTP	0.04769	0.047592	0.031613
ATP	10.18674	14.03449	10.95694
dGTP	0.017801	0.017076	0.011843
dCTP	0.017801	0.017076	0.011843
dATP	0.017103	0.016406	0.011379
dTTP	0.017103	0.016406	0.011379
C16:0	0.13747	0.098807	0.114874
C16:1	0.010623	0.003374	0.00421
C16:2	0.051864	0.022653	0.012029
C16:3	0.012497	0.022171	0.018043
C16:4	0.00125	0.00964	0.010826
C18:0	0.007498	0.026509	0.023456
C18:1	0.219952	0.136402	0.241175
C18:2	0.126222	0.077117	0.085404
C18:3	0.051864	0.075672	0.075781
C18:4	0.002499	0.005302	0.005413
ADPG	1.788051	2.086752	2.368441
Asp	0.227355	0.198168	0.096431
Asn	0.000199	0.000174	8.45E-05
Thr	0.114453	0.104282	0.039571
Ser	0.066115	0.094992	0.033226
Gln	0.00015	0.000139	7.03E-05
Glu	0.324899	0.300146	0.15188
Gly	0.018914	0.124881	0.138176
Ala	0.100857	0.158182	0.103952
Cys	0.019903	0.010238	0.013575
Val	0.205207	0.119612	0.056798
Met	0.023504	0.036243	0.01051
Ile	0.109856	0.0811	0.036649
Tyr	0.02996	0.06136	0.023386
Leu	0.222675	0.167428	0.071683
Phe	0.114037	0.109702	0.042935
Lys	0.250428	0.128822	0.170803
His	0.011765	0.048213	0.017687
Trp	7.32E-05	0.00015	5.71E-05
Arg	0.180285	0.282755	0.185818
Pro	0.07932	0.094576	0.04849
Glyc3P	0.624864	0.481984	0.601435

DXP	0.009614	0.015487	0.023476
ADP	10.18674	14.03449	10.95694

Supplementary Table 3. Reactions of central carbon metabolism in metabolic flux analysis.

**Glycolysis**
Gluc+ATP->G6P+ADP
F6P+ATP->FBP+ADP
FBP<=>DHAP+GAP
DHAP<=>GAP
GAP+NAD+ADP+Pi<=>3PG+ATP+NADH
3PG<=>PEP
PEP+ADP->PYR+ATP
G6P+ATP->ADP+ADPG
G6P+NADP->6PG+NADPH
ATP+CIT->ADP+OAA+AcCoA
**Pentose Phosphate Pathway**
6PG+NADP->Ru5P+CO2+NADPH
Ru5P<=>X5P
Ru5P<=>R5P
X5P+R5P<=>GAP+S7P
X5P+E4P<=>GAP+F6P
S7P+GAP<=>E4P+F6P
**TCA cycle**
PYR+NAD->AcCoA+CO2+NADH
AcCoA+OAA->CIT
CIT<=>ICIT
ICIT+NADP<=>AKG+CO2+NADPH
AKG+NAD->SucCoA+CO2+NADH
SucCoA+ADP+Pi<=>SUC+ATP
SUC+FAD<=>FUM+FADH2
FUM<=>MAL
MAL+NAD<=>OAA+NADH
**Glyoxylate Shunt**
ICIT->Glyox+SUC
AcCoA+Glyox->MAL
**Amphibolic Reaction**
MAL+NADP->PYR+CO2+NADPH
PEP+CO2->OAA+Pi
OAA+ATP->PEP+CO2+ADP
**Fatty acid Biosynthesis**
DHAP+NADH<=>Glyc3P+NAD
8AcCoA+7ATP+14NADPH->C16:0+7ADP+14NADP
C16:0+NADH+O2->C16:1+NAD
9AcCoA+8ATP+16NADPH->C16:0+8ADP+16NADP
C18:0+NADH+O2->C18:1+NAD
C18:1+NADH+O2->C18:2+NAD

C18:2+NADH+O2->C18:3+NAD
**Amino Acid Biosynthesis**
AKG+NADPH+NH3->Glu+NADP
Glu+ATP+NH3->Gln+ADP+Pi
Glu+2NADPH+ATP->Pro+2NADP+ADP+Pi
Glu+CO2+Gln+NADPH+Asp+AcCoA+5ATP->Arg+AKG+NADP+FUM+Ac+5ADP+5Pi
OAA+Glu->Asp+AKG
Asp+NH3+2ATP->Asn+2ADP+2Pi
PYR+Glu->Ala+AKG
3PG+Glu+NAD->Ser+NADH+AKG+Pi
Ser+THF<=>Gly+MEETHF
Gly+THF+NAD<=>CO2+MEETHF+NH3+NADH
Thr+NAD->Gly+AcCoA+NADH
Ser+AcCoA+SO4+3ATP+4NADPH->Cys+Ac+4NADP+3ADP+3Pi
Asp+PYR+Glu+2NADPH+ATP+SucCoA->LL-DAP+AKG+2NADP+ADP+Pi+Suc
LL-DAP->Lys+CO2
Asp+2NADPH+2ATP->Thr+2NADP+2ADP+2Pi
Asp+METHF+Cys+2NADPH+ATP+SucCoA->Met+PYR+2NADP+ADP+Pi+Suc+NH3+THF
2PYR+NADPH+Glu->Val+CO2+NADP+AKG
2PYR+AcCoA+Glu+NADPH+NAD->Leu+2CO2+AKG+NADP+NADH
Thr+PYR+Glu+NADPH->Ile+CO2+AKG+NADP+NH3
E4P+2PEP+Glu+NADPH+ATP->Phe+CO2+AKG+NADP+ADP+4Pi
E4P+2PEP+Glu+NADPH+NAD+ATP->Thr+CO2+AKG+NADP+NADH+ADP+4Pi
E4P+2PEP+R5P+Ser+Gln+NADPH+3ATP->Trp+CO2+Pyr+GAP+Glu+NADP+3ADP+6Pi
R5P+FTHF+Gln+Asp+5ATP+2NAD->His+2NADH+AKG+FUM+5ADP+6Pi+THF
**Oxidative Phosphorylation**
NADH+0.5O2+3ADP+3Pi->3ATP+NAD
FADH2+0.5O2+2ADP+2Pi->2ATP+FAD
**Transhydrogenation**
NADH+NADP<=>NADPH+NAD
**ATP hydrolysis**
ATP->ADP
**Transport**
Gluc.Ext->Gluc
CO2->CO2.Ext
O2.Ext->O2
**Nucleotide Biosynthesis**
2ATP+Gln+CO2->2ADP+Glu+CarP
R5P+ATP->PRPP+ADP
4ATP+CO2+PRPP+Asp+Gly+2Gln+2FTHF->FUM+2Glu+4ADP+IMP+2THF
3ATP+Gln+NAD+IMP->NADH+Glu+rGTP+3ADP

3ATP+Asp+IMP->FUM+rATP
CarP+Asp+NAD+PRPP+2ATP->2ADP+rUTP+CO2+NADH
ATP+Gln+rUTP->rCTP+ADP+Glu
NADPH+rCTP->dCTP+NADP
dCTP+METHF->dTTP+NH3+THF
NADPH+rATP->dATP+NADP
NADPH+rGTP->dGTP+NADP
**MEP pathway**
PYR+3PG->DXP+CO2
